# Structure of the dimeric *N*-glycosylated form of fungal β-*N*-acetylhexosaminidase revealed by computer modeling, vibrational spectroscopy, and biochemical studies

**DOI:** 10.1186/1472-6807-7-32

**Published:** 2007-05-17

**Authors:** Rüdiger Ettrich, Vladimír Kopecký, Kateřina Hofbauerová, Vladimír Baumruk, Petr Novák, Petr Pompach, Petr Man, Ondřej Plíhal, Michal Kutý, Natallia Kulik, Jan Sklenář, Helena Ryšlavá, Vladimír Křen, Karel Bezouška

**Affiliations:** 1 Laboratory of High Performance Computing, Institute of Systems Biology and Ecology of the Academy of Sciences of the Czech Republic and Institute of Physical Biology of USB, Zámek136, CZ-37333 Nové Hrady, Czech Republic; 2 Institute of Physics, Faculty of Mathematics and Physics, Charles University, Ke Karlovu5, CZ-12116 Prague2, Czech Republic; 3 Institute of Microbiology, Academy of Sciences of the Czech Republic, Vídeňská1083, CZ-14220 Prague4, Czech Republic; 4 Department of Biochemistry, Faculty of Science, Charles University, Albertov2030, CZ-12840 Prague2, Czech Republic

## Abstract

**Background:**

Fungal β-*N*-acetylhexosaminidases catalyze the hydrolysis of chitobiose into its constituent monosaccharides. These enzymes are physiologically important during the life cycle of the fungus for the formation of septa, germ tubes and fruit-bodies. Crystal structures are known for two monomeric bacterial enzymes and the dimeric human lysosomal β-*N*-acetylhexosaminidase. The fungal β-*N*-acetylhexosaminidases are robust enzymes commonly used in chemoenzymatic syntheses of oligosaccharides. The enzyme from *Aspergillus oryzae *was purified and its sequence was determined.

**Results:**

The complete primary structure of the fungal β-*N*-acetylhexosaminidase from *Aspergillus oryzae *CCF1066 was used to construct molecular models of the catalytic subunit of the enzyme, the enzyme dimer, and the *N*-glycosylated dimer. Experimental data were obtained from infrared and Raman spectroscopy, and biochemical studies of the native and deglycosylated enzyme, and are in good agreement with the models. Enzyme deglycosylated under native conditions displays identical kinetic parameters but is significantly less stable in acidic conditions, consistent with model predictions. The molecular model of the deglycosylated enzyme was solvated and a molecular dynamics simulation was run over 20 ns. The molecular model is able to bind the natural substrate – chitobiose with a stable value of binding energy during the molecular dynamics simulation.

**Conclusion:**

Whereas the intracellular bacterial β-*N*-acetylhexosaminidases are monomeric, the extracellular secreted enzymes of fungi and humans occur as dimers. Dimerization of the fungal β-*N*-acetylhexosaminidase appears to be a reversible process that is strictly pH dependent. Oligosaccharide moieties may also participate in the dimerization process that might represent a unique feature of the exclusively extracellular enzymes. Deglycosylation had only limited effect on enzyme activity, but it significantly affected enzyme stability in acidic conditions. Dimerization and *N*-glycosylation are the enzyme's strategy for catalytic subunit stabilization. The disulfide bridge that connects Cys^448 ^with Cys^483 ^stabilizes a hinge region in a flexible loop close to the active site, which is an exclusive feature of the fungal enzymes, neither present in bacterial nor mammalian structures. This loop may play the role of a substrate binding site lid, anchored by a disulphide bridge that prevents the substrate binding site from being influenced by the flexible motion of the loop.

## Background

Fungal β-*N*-acetylhexosaminidases catalyze the hydrolysis of chitobiose into its constituent monosaccharides. These enzymes are physiologically important during the life cycle of the fungus for the formation of septa, germ tubes and fruit-bodies [1-3]. These processes are important in control of fungal and insect pests [4] and are relevant to human diseases [5], lending considerable interest in the catalytic mechanism of these enzymes. The enzymes are also used in chemoenzymatic synthesis of biologically interesting oligosaccharides based on their effective transglycosylation of β-GlcNAc and β-GalNAc [6-9].

Crystal structures are known for several β-*N*-acetylhexosaminidases from the glycohydrolase 20 family including the monomeric bacterial enzymes from *Serratia marcescens *[10,11] and *Streptomyces plicatus *[12,13]. The catalytic domain of β-*N*-acetylhexosaminidase is an α/β TIM-barrel. Crystallization with substrate analogs showed the conserved residues Asp^539^-Glu^540 ^to be close to the binding site and thus predict them to play a key role in chitobiose hydrolysis with Glu^540 ^acting as a proton donor to the substrate, while Asp^539 ^restrains its acetamido group in a specific orientation by hydrogen bonding with N2 of the nonreducing sugar [11]. β-*N*-acetylhexosaminidase from *Streptomyces plicatus *has been co-crystallized with the cyclic intermediate analogue *N*-acetylglucosamine-thiazoline. The pyranose ring of the analogue is bound in the active site in a conformation close to that of a ^4^C_1 _chair. Within the substrate-binding pocket, Tyr^393 ^and Asp^313 ^appear important for positioning the 2-acetamido group of the substrate for nucleophilic attack at the anomeric center and for dispersing the positive charge distributed into the oxazolinium ring upon cyclization [12]. Experiments with two mutated forms of the enzyme (Asp^313^Ala and Asp^313^Asn) provided evidence that Asp^313 ^stabilizes the transition states, and assists to correctly orient the 2-acetamido group for catalysis [13]. Recently, the structure of dimeric human lysosomal β-*N*-acetylhexosaminidase has been solved providing new insight into the mechanism of Sandhoff disease [14]. Most mutations associated with late-onset Sandhoff disease reside near the subunit interface, and are thus proposed to interfere with the correct formation of the enzyme dimer [14].

The fungal β-*N*-acetylhexosaminidases are robust enzymes commonly used in our laboratories in chemoenzymatic syntheses of oligosaccharides [6,7,9]. We have previously reported the remarkable inducibility of a fungal β-*N*-acetylhexosaminidase from *Aspergillus oryzae *by GlcNAc [15]. This enzyme was purified to homogeneity from the culture medium, and its sequence was determined using both direct protein sequencing and DNA sequencing of a genomic clone containing the *hexA *gene [16]. In the present work, to initiate structural studies of this enzyme we performed sequence alignment and homology modeling, and constructed a molecular model of the enzyme and of its complex with the natural substrate chitobiose and with its non-cleavable analog GlcNAcβ1→4ManNAc [17]. Several experimental approaches provide experimental verification of the overall features suggested by the model. Disulfide bridging, secondary structure, and the mode of subunit assembly were determined experimentally and correlated with the model. We also constructed a model of the *N*-glycosylated enzyme, and compared it with kinetic properties of the native and deglycosylated enzymes.

## Results

### Molecular models of β-*N*-acetylhexosaminidase

**Figure 1:**
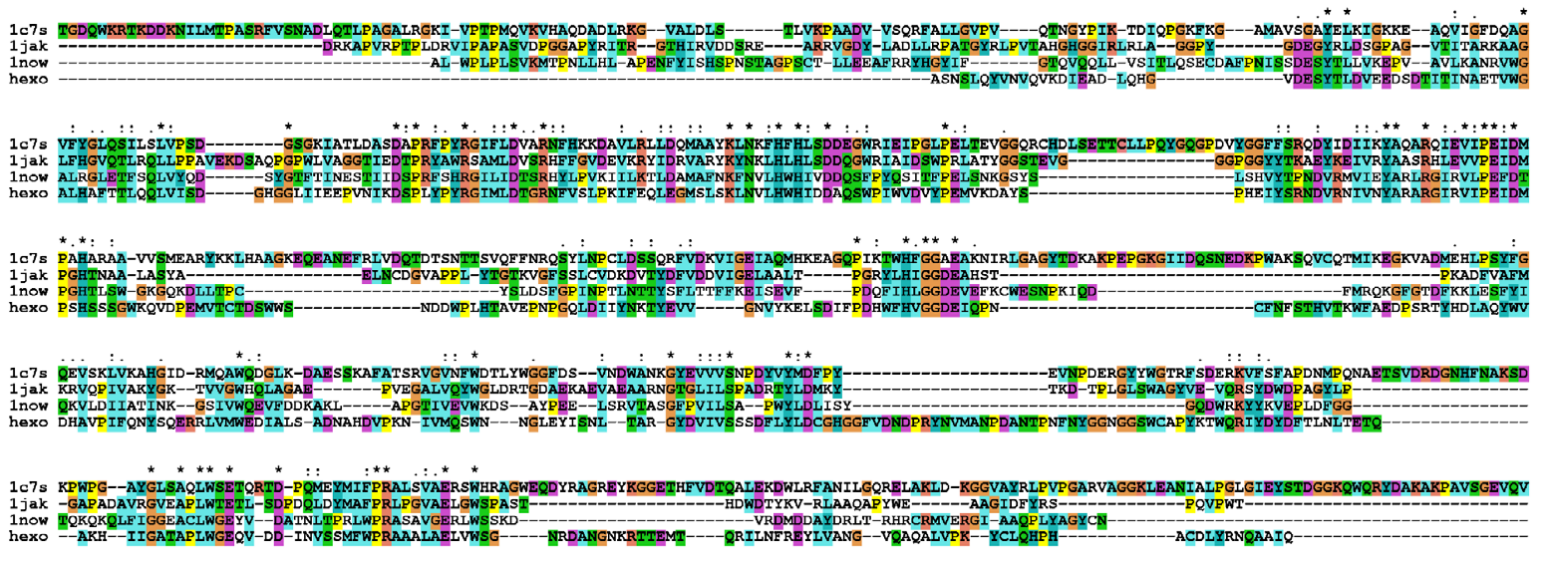
**Primary sequence alignment**. Alignment of the amino acid sequence of β-*N*-acetylhexosaminidase from *Aspergillus oryzae *with the three hexosaminidases having the solved three-dimensional structure (1c7s: *Serratia marcenscens*, 1jak: *Streptomyces plicates*, 1now: *Homo sapiens*).

**Figure 2:**
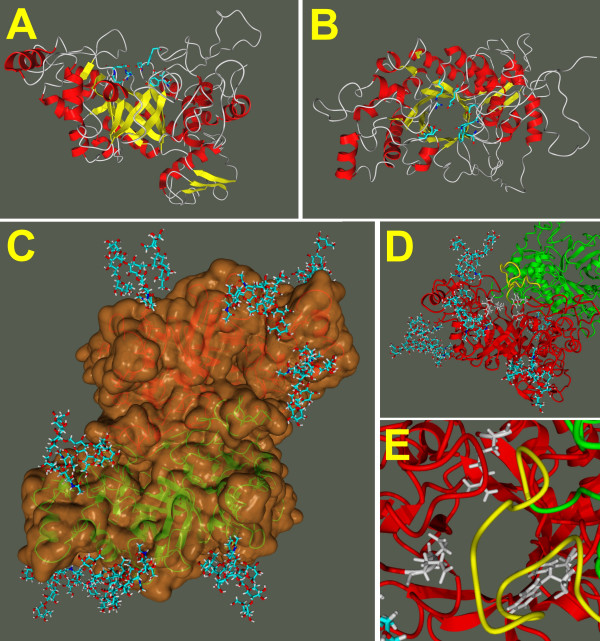
**Molecular models of β-*N*-acetylhexosaminidase from *Aspergillus oryzae***. The models show the shape of the catalytic subunit from a side view (A) and a top view (B) with the active site at the C-terminal face of the (β, α)_8_-barrel, and the arrangement of these subunits in the fully *N*-glycosylated dimer (C). The large flexible loop (D: side view, E: top view, shown in yellow) of the green monomer is just about 1 nm above the active site residues (shown in grey) of the red monomer.

**Figure 3:**
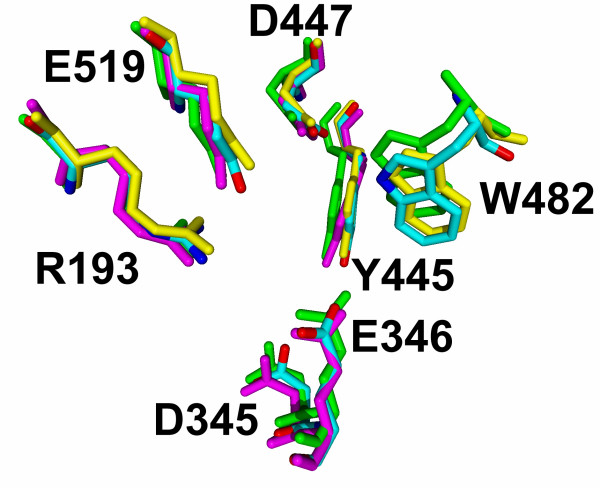
**Overlay of the active site residues of the refined homology model with the crystal structures**. Active site residues of the homology model of the complete monomer refined by 20 ns of molecular dynamics in a NPT ensemble, are shown in an overlay with PDB entry: 1c7s: *Serratia marcenscens *(yellow), 1jak: *Streptomyces plicates *(green), 1now: *Homo sapiens *(magenta). In the human structure the tryptophane residue is not conserved and thus missing in the structure, and in the *Serratia marcenens *structure the aspartic acid is mutated to alanine. The overlay shows clearly a spatial alignment with our calculated and refined structure.

We have used the primary structure of the fungal β-*N*-acetylhexosaminidase determined in our laboratory [16] to perform homology modeling using the solved structures of these enzymes from *Serratia marcescens *[11], *Streptomyces plicatus *[18] and *Homo sapiens *[14,19]. Alignment of the amino acid sequences of the fungal enzyme and these three structurally solved enzymes reveals both areas of extensive amino acid similarity and segments that appear unique to the fungal enzyme (Fig. 1). The shown alignment is the result of a combination of a structural alignment of the three crystal structures and the sequence alignment generated with ClustalX, to exclude misalignment in sequence variable regions, as especially the chitobiase from *Serratia marcescens *has additional domains. Starting from this alignment we created a structural model of the catalytic subunit including the small *N*-terminal zincin-like domain using a restraint-based comparative modeling approach. In this model we grouped the six cysteins into three pairs according to the closest distance. The three cysteine pairs Cys^290^-Cys^351^, Cys^448^-Cys^483 ^and Cys^583^-Cys^590 ^were adjusted into the model by repeating the modeling procedure with the additional restraints as an input for Modeller. The final model had 82.8% of residues in the most favored regions of the Ramachandran plot and an acceptable overall geometry, both determined with the ProCheck program. The overall *g*-factor of the structure obtained showed a value of -0.22. The *g*-factor tries to quantify the overall geometry and its value should be above -0.5; values below -1.0 may indicate a wrong structure. With respect to the general shortcomings of homology modelling especially in the loop and sequence variable regions, we decided to solvate the homology structure in SPC water to refine it by 20 ns of molecular dynamics in a NPT ensemble. According to the root mean square deviation of the C_α _atoms the structure gets after 7 ns into an equilibrium state oscillating around a fixed value with a deviation of ± 0.3 Å. The resulting catalytic subunit has a kidney-shaped structure with approximate dimensions of 6.8 × 5.8 × 5.6 nm (Fig. 2A). All amino acids involved in catalysis are concentrated in the central TIM barrel, with the catalytic glutamic acid on the upper border (Fig. 2B, center). An overlay with the corresponding amino acids of the three template structures with our final model shows that their position is conserved among the available structures and that the active site in our model structure was stable during the molecular dynamics refinement of the overall structure (Fig. 3).

**Figure 4:**
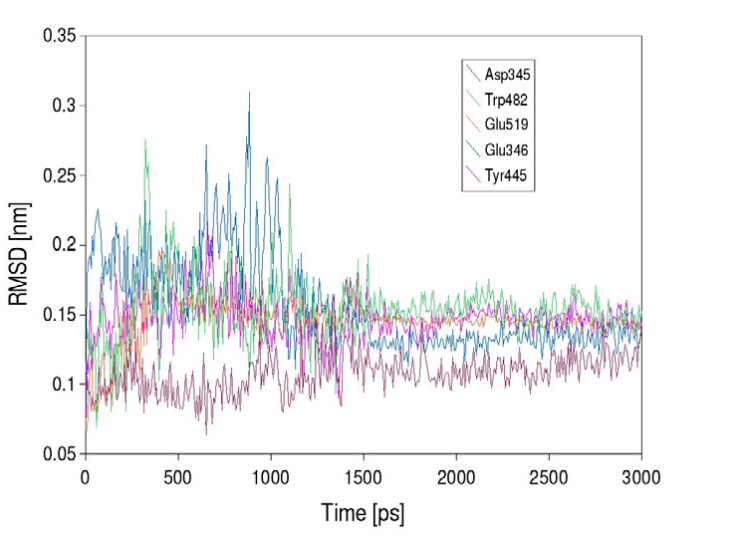
**Root mean square deviation of the active stite residues after substrate docking**. The root mean square deviation of the main amino acids in the active site was monitored for 3 ns.

**Figure 5:**
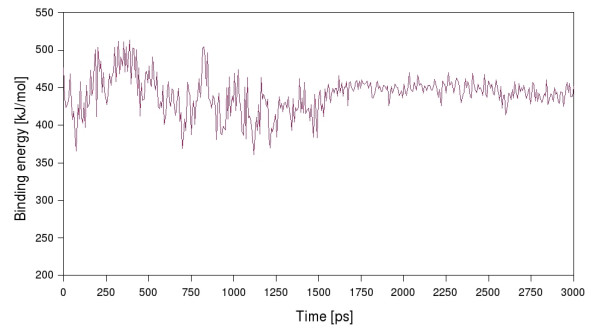
**Binding energy of chitobiose during molecular dynamics**. Chitobiose was docked into the active site of the homology model of the complete monomer, that was solvated in SPC water and refined by 20 ns of molecular dynamics in a NPT ensemble to equilibrate the homology structure. Behaviour of the substrate in the active site was monitored for 3 ns and the binding energy showed an average value of 447.1 kJ/mol in the time period of 1.6 ns to 3 ns.

**Figure 6:**
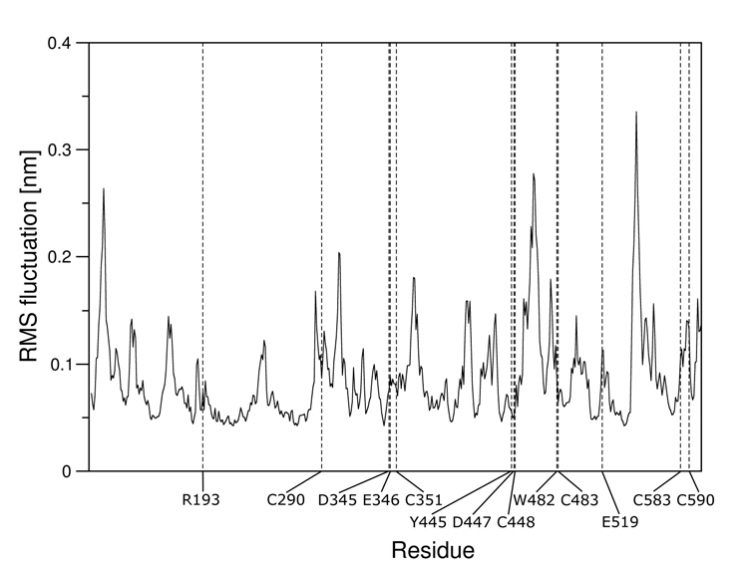
**Protein flexibility**. Root mean square fluctuation of β-*N*-acetylhexosaminidase from *Aspergillus oryzae *during the last 10 ns of MD simulation at 300 K. Amino acids in the active site and cysteins envolved in the formation of disulphide bridges are labeled.

Docked into the binding site, the natural substrate *N, N'*-diacetylchitobiose, participates in the hydrogen bonding between Asp^345 ^and Tyr^445 ^with the acetamido group of the non-reducing GlcNAc moiety. The non-reducing end of the disaccharide is locked into the active site owing to the hydrogen bonds of Arg^193^, Asp^447^, Trp^482 ^and Glu^519 ^with the C4 and C5 OH groups. The reducing end of the disaccharide is stabilized by a π-π interaction with the aromatic ring of Trp^482^. After the substrate being docked and the complex being solvated it shows an initial equilibration phase with rather high fluctuations of the observed binding energies and quite large changes in root mean square deviation of the binding site residues (Fig. 4). However, after 1.6 ns of MD simulation the system seems to have established the binding energy gets stable with only minor fluctuation and the root mean square deviation of the binding site residues arrives back at a stable value for each of the residues that corresponds to the concrete amino acids rigidity (Fig. 5). The mean value of the binding energy over the time interval from 1.6 ns to 3 ns is 447.1 kJ/mol [see Additional file 1]. The root mean square fluctuation of the protein is measured during the last 10 ns of the 20 ns MD simulation, as the structure is at that time already several nanoseconds in a equilibrium state. Figure 6 shows all active site residues belonging to the less flexible part of the protein with minimum fluctuation in time. The root mean square fluctuation of Arg^193^, Tyr^445^, Asp^447 ^and Trp^482 ^describes these residues as the most rigid part of the protein and thus these amino acids should play the key role for substrate specificity. Asp^345 ^and the catalytic residue Glu^346 ^show a little less rigidity which can be interpreted that they must be able to orient themselves to the *O*-glycosidic bond after substrate binding.

Since initial biochemical characterization of β-*N*-acetylhexosaminidase from *Aspergillus oryzae *indicated it to be a dimer, we used the physiologically relevant dimeric crystal structure of human β-*N*-acetylhexosaminidase [14] (pdb ID: 1o7a) to model the dimeric fungal enzyme according to the physiolocially valid dimerization interface I found in the crystal structure (Fig. 2C). Similar like in crystal structure of human β-*N*-acetylhexosaminidase, the proposed dimer interface is formed mainly by loop regions at the C-terminal face of the (β, α)_8_-barrel, as is the active site. Furthermore, the large flexible loop residues anchored by the disulphide bridge of Cys^448 ^with Cys^483^, an exclusive feature of the fungal enzymes, contributes to the dimer interface.

Detailed examination of the dimer contact surface reveals a buried surface area per monomer of 2373 Å^2 ^and 64 residues containing atoms closer than 3.6 Å to atoms of the interface partner with 19 residues being hydrophobic (Ile, Leu, Val, Met, Tyr, Phe and Trp). We were able to identify 39 hydrogen bonds between both monomers, with the first monomer accepting 22 and donating 17. The presence of two Arg, three His, three Glu and 4 Asp explains the importance of ionic interactions between of Arg and His residues of one subunit and Asp and Glu residues of the opposite subunit. The model thus indicates that the β-*N*-acetylhexosaminidase dimer may be a reversible function of pH, with acidic environments favoring dissociation into subunits. Indeed, down to pH3.5 the enzyme exists exclusively in the dimeric form, but at pH2.5 it exists only as monomeric, but enzymatically active, subunits as revealed by gel filtration and enzyme assay [20]. When the dissociated enzyme is titrated back to pH5.0, after a short incubation the dimer forms to the original extent [20]. This result also indicates contact areas that are easily re-established depending on the ionization state of residues with pK_a _values near pH3. The role of the ionic pairs of the above type in the formation the dimeric structure of hexosaminidases is well established. In human hexosaminidase B there is Asp^494^-Arg^533 ^at the interface between the catalytic subunits in the dimer [14] (Asp^522 ^and His^586 ^in our structure). Notably, since in human hexosaminidases the dimerization is essential for the full catalytic activitity, mutations in the above "interface" amino acids are among the well documented human mutations that have been found in the less severe (late onset) forms of Sandhoff [14] and Tay-Sachs [18,21] diseases. Interestingly, in the dimeric structure the large flexible loop of the opposite monomer is about 1 nm above the C-terminal face of the (β, α)_8_-barrel, and the active site giving the impression of a substrate binding site lid (Fig. 2D, E).

**Figure 7:**
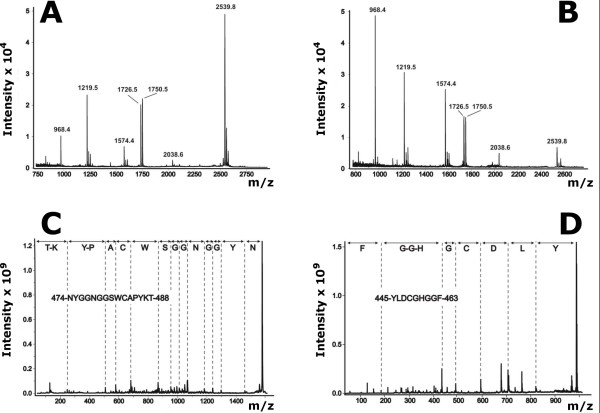
**Examination of the status of cysteines in the β-*N*-acetylhexosaminidase molecule**. Cystic peptides were analyzed by MALDI mass spectrometry either before (A) or after (B) the addition of DTT to the sample. The individual assignments were further confirmed by PSD MALDI mass spectrometry of peaks with *m*/*z *1574.4 (C) and 968.4 (D).

Six putative sites of *N*-glycosylation are present in the primary structure of the fungal enzyme [20]. Digestion of β-*N*-acetylhexosaminidase with *N*-glycanase results in significant mobility shift on SDS-PAGE (Figure 7A), indicating that one or more glycosylation sites are used on the secreted enzyme. In order to reveal the details of these important structural modifications, we have performed a detailed analysis of the actual occupaccy of all these sites using the standard *N*-glycanase/H_2_^18^O technique [22,23] and mass spectrometry which revealed that all six sites of *N*-glycosylation were indeed used in the actual hexosaminidase preparation. This conclusion was further supported by complete proteolytic digestion of hexosaminidase followed by isolation of the glycopeptides on immobilized plant lectin Concanavalin A, and identification by these glycopeptides by mass spectrometry. Glycopeptides that covered all six sites of *N*-glycosylation could be identified on the mass spectra (results not shown).

The fact that all the glycopeptides described above could be efficiently recovered on the immobilized Concanavalin A provided a strong indication for the high-mannose type oligosaccharides being present at the individual *N*-glycosylation sequences. In order to support this indication, the oligosaccharides were released by *N*-glycanase and analyzed using a combination of Dionex oligosaccharide profiling, exoglycosidase digestion, and mass spectrometry as described in the Experimental section. When we performed this analysis on oligosaccharides released from the entire hexosaminidase, we obtained the overall glycosylation of the entire enzyme indicating the hexamannosyl oligosaccharide M6 as the predominant structure (Table 1, lane Asn^all sites^). This overall glycosylation profile was very similar to the one we found on the two particular sites of glycosylation Asn^427 ^and Asn^499 ^(Table 1). As the experimental results show a M6 structure as the predominant oligosaccharide, we choose this structure as the oligosaccharide to be attached to the molecular model of β-*N*-acetylhexosaminidase. In order to get a preliminary insight into the structural arrangement, and possible biological role(s) of *N*-glycans in the fungal β-*N*-acetylhexosaminidase, we have sterically fitted and covalently linked the most prevalent (M6) oligosaccharide to each site of glycosylation on our model structure. This gave rise to the model of a fully glycosylated enzyme shown as the most prevalent glycoform in Fig. 2C. Although the analysis of the actual glycoforms would be a much more demanding task, the model shown in Fig. 2C illustrates nicely the surface exposure of the individual oligosaccharides protecting the enzyme dimer from the effects of the extracellular environment.

**Table 1:** Oligosaccharide composition at the individual sites of *N*-glycosylation

**Site**	Oligosaccharides containing the indicated number of mannoses (% of total)							
	M4	M5	M6	M7	M8	M9	M10	M11

Asn^427^	--	19	30	21	19	11	--	--
Asn^499^	19	26	14	14	24	3	--	--
Asn^all sites^	9	14	26	23	15	7	4	2

### Evidence for the status of cysteines using protein chemistry and mass spectrometry

The fungal β-*N*-acetylhexosaminidase is secreted into the extracellular environment, indicating the possibility for forming disulfide bonds. Since there are six cysteines in the primary structure of β-*N*-acetylhexosaminidase, the status of their sulfhydryl groups was determined by mass spectrometry [24]. It has been found that the experimentally verified arrangement of the disulfide bridges corresponds exactly to that anticipated by the molecular model (see above). Moreover, these results were further confirmed by differential mapping of pepsin peptides with and without DTT reduction. Using this technique reverse phase HPLC identified only one peak with significant shift in the retention time. One of the components of this chromatographic peak was the peptide with *m*/*z *2538.8 (Fig. 7A). Significant decrease in its intensity with concomitant intensity increase of peaks at *m*/*z *1574.0 and 968.2 (Fig. 7B), observed upon addition of DTT, indicates that this peptide consists of two disulfide linked peptides. These were identified by PSD measurement as peptides Tyr^445^-Phe^453 ^and Asn^474^-Thr^488 ^containing the cysteines 448 and 483, respectively. This confirms the identification of the disulfide bond between Cys^448 ^and Cys^483^.

In the model structure without disulfide bridges, Cys^483 ^is 6.2 Å away from Cys^448^. The formation of the disulfide bond in the model is leading to a somewhat different position of one of the loops, easily accommodating the experimentally determined disulfide bridge with only a relatively minor change in the overall structure. As Asp^447 ^and Trp^482 ^are both amino acids forming the active site, the flexible loop between Cys^448 ^and Cys^483 ^is right beside the substrate-binding site. The behavior of this loop measured by root mean square fluctuation during the last 10 ns MD simulation shows that it is highly flexible (Fig. 6), which may be functionally important. The nearby disulfide bond thus stabilizes not only this loop but also the active site.

### The effect of deglycosylation on enzyme activity and stability

**Figure 8:**
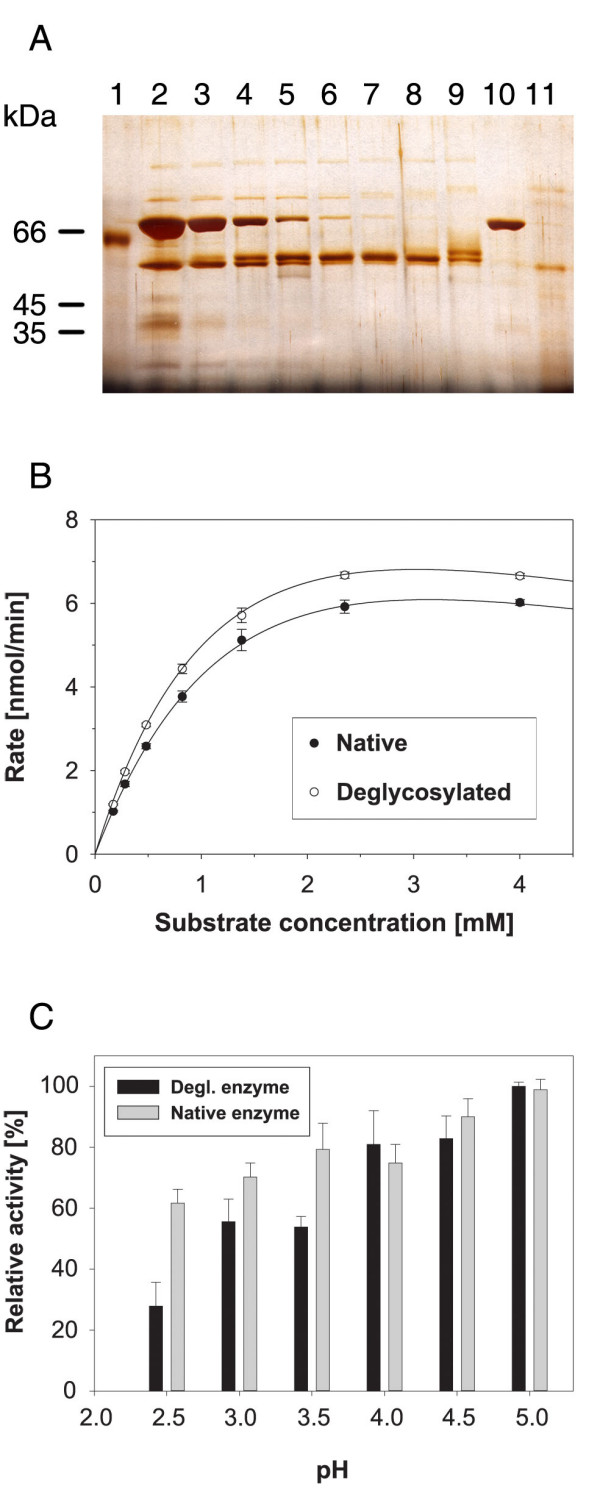
**Effect of glycosylation on enzymatic activity and stability of β-*N*-acetylhexosaminidase**. (A) deglycosylation of the enzyme by endoglycosidase H and *N*-glycanase under native conditions. β-*N*-acetylhexosaminidase (0.1 μg, lane 1) was deglycosylated using 10 U (lane 2), 5 U (lane 3), 2 U (lane 4), 1 U (lane 5), 0.5 U (lane 6), 0.2 U (lane 7), 0.1 U (lane 8), or 0.05 U (lane 9) of endoglycosidase H (Endo H_f_). Lane 10 contains Endo H_f _control, and lane 11 β-*N*-acetylhexosaminidase (0.1 μg) deglycosylated by 0.1 U of *N*-glycanase. (B) comparison of the enzymatic parameters of native and deglycsylated β-*N*-acetylhexosaminidase. (C) effect of β-*N*-acetylhexosaminidase deglycosylation on the stability of the enzyme under various pH values. The average values from triplicate determinations with the standard error indicated by the error bars are shown.

Preliminary deglycosylation experiments indicated the possibility to deglycosylate the enzyme under native conditions (Fig. 8A, *cf*. lane 1 and 11). We evaluated several protocols for deglycosylation using *N*-glycanase or endoglycosidase *H *under conditions compatible with optimal stability of the deglycosylated hexosaminidase (Fig. 8A). The deglycosylated enzyme remains fully active (Fig. 8B), and displays kinetic parameters indistinguishable from those of the native enzyme (K_m _= 0.71 mM and V_max _= 2.16 nmol/min *vs*. K_m _= 0.45 mM and V_max _= 1.55 nmol/min for the native form).

To probe the effects of deglycosylation on enzyme stability, native and deglycosylated enzymes were incubated at a range of pH values. No significant activity differences were found at alkaline or neutral pH. However, the deglycosylated enzyme is significant less stable than the native enzyme at pH values below 4. The activity of the deglycosylated enzyme after incubation at pH3.5 and 3.0 is about 75% of the native enzyme, and at pH2.5 the deglycosylated enzyme is only half as active as the native enzyme (Fig. 8C). This result suggests that *N*-linked oligosaccharides may be important for the stabilization of β-*N*-acetylhexosaminidase at low pH.

### Structure and stability determined by vibrational spectroscopy

**Figure 9:**
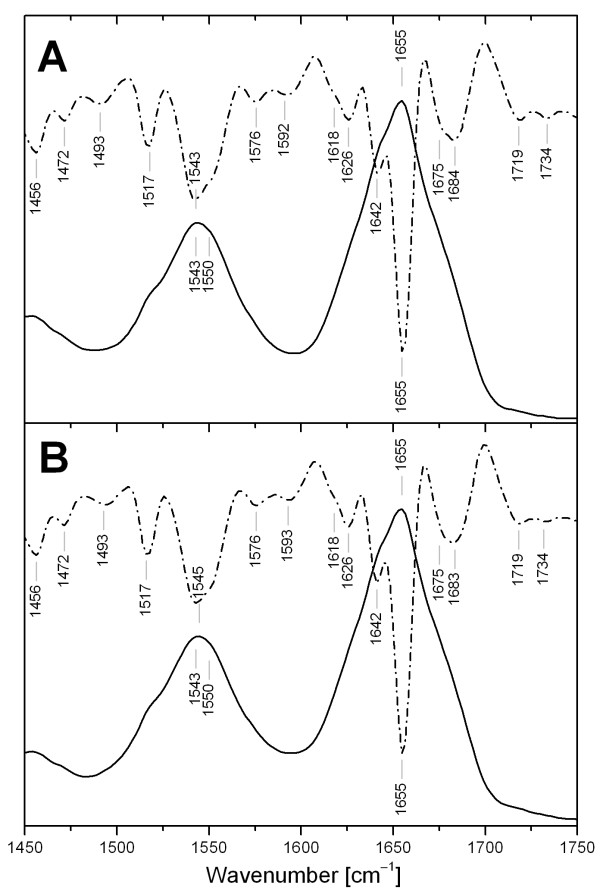
**Infrared spectra of β-*N*-acetylhexosaminidase**. Comparison of the infrared spectra in the amide I and II regions of β-*N*-acetylhexosaminidase (A) and β-*N*-acetylhexosaminidase deglycosylated (B). The solid curves represent the original spectra while the dashed curves are associated with the second derivative (15 pts) of the spectra.

Infrared spectra of β-*N*-acetylhexosaminidase are characterized by two major bands at 1655 cm^-1^, and 1543 cm^-1 ^(Fig. 9A), associated with the amide I and II vibrations, respectively. The position and shape of these bands are sensitive to protein conformation and secondary structure content. The second derivative, which can identify overlapping components, reveals three major bands in the amide I region. The largest component at 1655 cm^-1 ^belongs to α-helical and disordered conformations [25,26]. Two other components at 1642 cm^-1 ^and 1626 cm^-1 ^are assigned to β-sheets [25,26]. The unresolved band at 1684 cm^-1 ^indicates the presence of antiparallel β-strands [27], and the second part of the unresolved band at 1675 cm^-1 ^corresponds to β-turns [25,26]. Characteristic side chain absorption of Tyr and Phe is observable at 1517 cm^-1 ^and 1493 cm^-1^, respectively [26]. The FTIR spectrum of β-*N*-acetylhexosaminidase after deglycosylation (Fig. 9B) shows no significant shifts with respect to the native protein.

**Figure 10:**
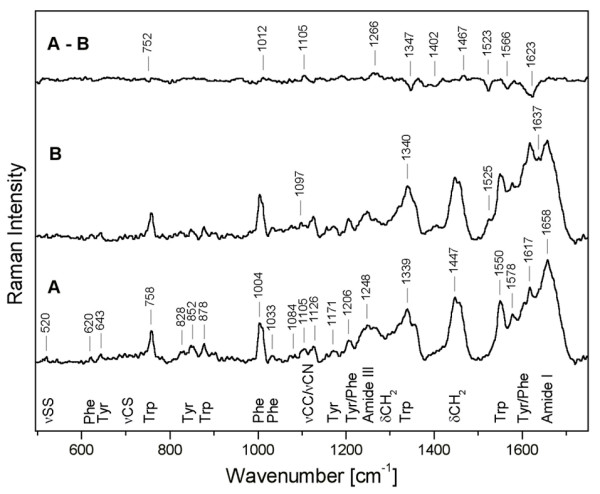
**Raman spectra of β-*N*-acetylhexosaminidase**. Raman spectrum of the native β-*N*-acetylhexosaminidase (A), of the enzyme deglycosylated by endoglycosidase H (B), and the differential spectrum of the two enzymes (A-B). The assignment of the bands is discussed in the text (ν corresponds to stretching and δ to bending vibrations).

Raman spectroscopy (Fig. 10) confirms the presence of disulfide bonds by the band at 520 cm^-1 ^due to the S-S stretching vibrations. The frequency of this band is sensitive to the local conformation of a disulfide bridge. The band at 520 cm^-1 ^corresponds to the sulfide bridges in a conformation close to *gauche-gauche-trans *(GGT) [28]. The bands in the region of stretching CS vibrations 700–745 cm^-1 ^can be hardly resolved due to presence of three disulfide bridges and their probable flexibility with respect to ν CS vibrations. The intensity ratio of the tyrosine Fermi resonance doublet (828 cm^-1 ^and 852 cm^-1^; I_852_/I_828 _= 1.6) indicates that the tyrosine OH group acts as an acceptor of H-bonds suggesting that some of the tyrosines are solvent exposed [29]. The Raman spectrum after deglycosylation (Fig. 10B) shows significant changes with respect to native protein (Fig. 10A) that are clearly revealed by the difference spectrum (A-B). Almost all marker bands of aromatic side chains are affected by deglycosylation, although the secondary structure was not affected, as discussed below. Thus, changes in the Raman spectrum can be attributed to changes in environment of the residues, presumably due to unmasking of large surface areas of the protein by deglycosylation.

The FTIR amide I and II bands and the Raman amide I band were analyzed by LSA (Table 2) to quantify the secondary structure content of β-*N*-acetylhexosaminidase and its deglycosylated form. Deglycosylation of the protein leads to small changes in secondary structure, as estimated by LSA, connected with slight decrease of β-sheets (about 3%) and slight increase of α-helices – probably on the periphery of the protein. The LSA methods employs two types of α-helical structure for Raman spectra: short helical segment up to eight residues called monohydrogen-bonded or disordered, and helical segments bracketed by at least four residues on each side named bihydrogen-bounded or ordered. Recently we proposed that the vibrations could be affected by oligomerization or complex formation [30], therefore the amount of disordered α-helix (about 15%) was included to other structure in Table 2. If we agree with interpretation that the signal from disordered α-helix is caused mostly by dimerization of β-*N*-acetylhexosaminidase then carbohydrate moiety plays important role in the dimer formation. The lower stability of the deglycosylated enzyme upon prolonged incubations at acidic pH (see Fig. 8C) may then be interpreted as the gradual decomposition of the enzyme dimer into the monomeric units that is fully reversible for the native but not for the deglycosylated enzyme.

**Table 2:** Secondary structure estimation (in %) of β-*N*-acetylhexosaminidase by FTIR and Raman spectroscopy compared with the model structures

Method	Secondary structure estimation				
	α-Helix	β-Sheet	β-Turn	Bend	Other

Model	32	15	14	14	25
FTIR-LSA^a^	31	19	14	14	22
Raman-LSA^b^	31	26	15	--	26

^a ^Least-squares analysis of FTIR amide I and II bands according to [55]. ^b ^Least-squares analysis of Raman amide I band according to [54].

## Discussion

We describe here results of structural analysis of dimeric, fully *N*-glycosylated, fungal β-*N*-acetylhexosaminidase isolated from the culture medium of the public collection strain of *Aspergillus oryzae*. The unique biology [15] and important biotechnological applications of this enzyme [8,9,17] stimulated this work as a prerequisite for structure-function studies and enzyme engineering. To create a conceptual basis for the design of structural experiments, the amino acid sequence of the *Aspergillus *β-*N*-acetylhexosaminidase determined in our laboratory [16] was used to generate a molecular model of the catalytic subunit based on sequence homology to three solved β-*N*-acetylhexosaminidase structures [10,12,14]. Our major experimental efforts then have been directed towards understanding the principal differences between the intracellular bacterial enzymes and the eukaryotic enzymes that have to undergo posttranslational modifications to survive better under the more aggressive conditions in lysosomes and the extracellular environment in which they function. In particular, we investigated how enzyme dimerization, disulfide bonding, and glycosylation participate in enzyme stabilization.

Multimerization of enzymes represents one strategy for catalytic subunit stabilization that is often used, for example, in thermophilic organisms. Consistent with this trend, the intracellular bacterial β-*N*-acetylhexosaminidases are monomeric whereas the extracellular secreted enzymes of fungi and humans occur as dimers. Dimerization of the fungal β-*N*-acetylhexosaminidase appears to be a reversible process that is pH dependent. This result provides strong support for the participation of titratable functional groups at the subunit interface as predicted by the constructed dimeric structure. The experimental finding that oligosaccharide moieties may also participate in the dimerization process may represent a unique feature of the exclusively extracellular enzymes.

Formation of disulfide bridges is another common way of stabilizing proteins that are secreted into the oxidative extracellular environment. The disulfide bridges detected experimentally are in complete accordance with the molecular model. One disulfide may stabilize a hinge region in a small flexible loop close to the substrate binding site. This loop is an exclusive feature of the fungal enzymes, neither present in bacterial nor mammalian structures. We hypothesize that this loop may play the role of a substrate binding site lid, as has been found previously in several enzymes including other TIM barrels [31-34], anchored by a disulfide bridge that prevents the substrate binding site from being influenced by the flexible motion of the loop. This substrate binding site lid is formed in the dimeric structure by the large flexible loop of the opposite monomer and is close above the C-terminal face of the (β, α)_8_-barrel, and the active site thus indicating the necessity of a functional dimer for full enzyme function.

Yet another posttranslational modification known to stabilize proteins is *N*-glycosylation. However, experimental investigation of its role in enzyme function and stabilization is technically difficult, and only a few studies report detailed experimental analysis [22,35]. In the case studied here we had the rare possibility to fully de-*N*-glycosylate the enzyme under native conditions. To probe the role of extensive glycosylation in the fungal β-*N*-acetylhexosaminidase experimentally we used two deglycosylation methods, which are based on the use of either *N*-glycanase or endoglycosidase H. The former enzyme is an asparagines amidase that cleaves the *N*-glycosidic (amide) bond between the asparagines and the oligosaccharide chains. This cleavage gives rise to the free β-glycosylamine, and aspartic acid that remains in the protein in the original position of the asparagines. On the other hand, the latter enzyme is a typical endoglycosidase that cleaves a glycosidic linkage between the two GlcNAc residues forming the chitobiose core of the *N*-linked oligosaccharide. Such a cleavage leaves the *N*-glycosidic bond on asparagines, and the proximal GlcNAc intact.

We employed endoglycosidase H digestion which allows to efficiently remove the mannose branches while retaining the core GlcNAc residues to avoid the potential dangers of complete protein deglycosylation that we have previously reported [36]. Deglycosylation had only limited effect on enzyme activity, but it significantly affected enzyme stability in acidic conditions. This role of *N*-glycosylation is consistent with the observation that the enzyme completely deglycosylated by *N*-glycanase has low stability and tends to precipitate.

## Conclusion

In summary, we have shown that complementary biochemical and biophysical methods provided structural insight into critical features of fungal β-*N*-acetylhexosaminidase, even in the absence of data from protein crystallography or nuclear magnetic resonance. The results reported here also contain useful information for designing experiments that will use high resolution methods of protein structure analysis. Dimerization of the fungal β-*N*-acetylhexosaminidase appears to be a reversible process that is strictly pH dependent. Oligosaccharide moieties may also participate in the dimerization process which might represent a unique feature of the exclusively extracellular enzymes. Deglycosylation had only limited effect on enzyme activity, but it significantly affected enzyme stability in acidic conditions. Dimerization and *N*-glycosylation are the enzyme's strategy for catalytic subunit stabilization. One disulfide bridge, neither present in bacterial nor mammalian structures, anchors a flexible loop close to the active site that may play the role of a substrate binding site lid in the physiologically relevant dimer.

## Methods

### Molecular modeling

The molecular model of β-*N*-acetylhexosaminidase was generated by a combination of energetic and homology modeling. In a first step three homologs from the glycohydrolase family 20 were extracted from the Protein Databank [37] – PDB entry: 1c7s: *Serratia marcenscens *[11], 1jak: *Streptomyces plicates *[18], 1now: *Homo sapiens *[19]. These proteins show a primary sequence identity about 30% and a homology around 45% (*Serratia marcenscens *32/48%, *Streptomyces plicates *27/42%, *Homo sapiens *31/49%) making homology modeling possible. A structural alignment was generated in SwissPDBViewer [38] showing a root mean square deviation ~1.4 Å between the three structures. Then the complete primary sequence of β-*N*-acetylhexosaminidase from *Aspergillus oryzae *[16] was aligned with the three homologs in ClustalX [39] keeping the positions of the structural alignment conserved (see Fig. 1). The three-dimensional model constituted by all non-hydrogen atoms was built and examined by the Modeller6 package [40]. All three disulfide bridges were created and refinement was achieved through algorithmic analysis and minimization with the Tripos force field in the Sybyl/Maximin2 (Tripos). Hydrogen atoms were added and the model of the generated structure was minimized to convergence of the energy gradient less then 0.01 kcal/mol using the Powell minimiser. The minimization included electrostatic interactions based on Gasteiger-Hückel partial charge distributions using a dielectric constant with a distance dependent function ε = 4*r *and a non-bonded interaction cut-off of 8 Å. The tertiary structure model was checked with Procheck [41].

The final proposed model was solvated in simple point charge water and four chloride counter-ions were added. The production runs were preceded by short equilibration runs of altogether 250 ps with positional restraints applied on the protein atoms to allow the solvent to relax and 250 ps of protein relaxation without positional contraints and a timestep of 2 fs. The simulation box was sized 1 nm in each direction from the protein surface and filled with 18950 water molecules to give a system of 62049 atoms. In a first step the system is minimized by 500 steps of steepest descent minimization, followed by 20 ps of solvent relaxation with a 1 fs timestep. Chloride ions were added by replacing four water molecules to neutralize the systems, followed by 30 ps of ion and solvent relaxation with a 1 fs timestep and 200 ps with a 2 fs timestep. The following production MD simulations, without any restraints, were 20 ns long and were run with GROMACS 3.2 [42,43] using the gmx force field, with a 5 fs time step (which is possible because dummy hydrogens are used). SETTLE (for water) and LINCS were used to constrain covalent bond lengths, and long-range electrostatic interactions were computed with the Particle-Mesh Ewald method. The temperature was kept at 300 K by separately coupling the protein and solvent to an external temperature bath (τ = 0.1 ps) [44]. The pressure was kept constant at 1 bar by weak coupling (τ = 1.0 ps) to a pressure bath. The protein proved to be stable during simulation.

The three dimensional structure of the *N*-linked complex glycan (M6 oligosaccharide structure) was calculated with Sweet [45] and the carbohydrates were sterically fitted by visual analysis and covalently attached to the molecular model of β-*N*-acetylhexosaminidase in Sybyl. The dimeric structure was created by fitting two monomeric structures onto the dimeric structure from *Homo sapiens *within YASARA [46]. Then the second monomer was placed in a distance of about 1 nm away from its final position to avoid sterical conflicts of the large flexible loop with the first monomer, followed by 2000 steps of simulated annealing minimization. The second monomer was such moved in 0.1 nm steps to its final position, each step followed by another 2000 steps of simulated annealing minimization.

Chitobiose was build in YASARA, forcefield parameters were assigned using the AutoSMILES approach [44], in a first step YASARA calculated semi-empirical AM1 Mulliken point charges that were corrected by assignment of AM1BCC atom types and improved AM1BCC charges by fragments of molecules with known RESP charges, to closer resemble RESP charges. Corresponding bond, angle and torsion potential parameters are taken from the General AMBER force field. For the docking experiments our model structure was fitted onto the crystal structure of 1qbb [10], a bacterial chitobiase complexed with *N*, *N*'-diacetylchitobiose. Chitobiose was placed in an arbitrary position according to the ligand co-ordinates in the bacterial chitobiase complex. Exact positioning of the ligand was done by a two-step procedure, energy minimization followed by a molecular dynamics. The ligand-protein system was minimized by 2000 steps followed by a 3 ns MD simulation in aqueous solution using the YAMBER2 force field [46]. The protein structure was placed into a box, which was 1 nm larger than the protein along all three axes. The box was filled with TIP3P water, sodium ions were iteratively placed at the coordinates with the lowest electrostatic potential until the cell was neutral. Molecular dynamics simulations were run with YASARA, using a multiple time step of 1 fs for intra-molecular and 2 fs for intermolecular forces. A 1.2 nm cut-off was taken for Lennard Jones forces and the direct space portion of the electrostatic forces, which were calculated using the Particle Mesh Ewald method [47] with a grid spacing 0.1 nm, 4^th ^order B-splines, and a tolerance of 10^-4 ^for the direct space sum. The simulation of interaction was then run at 298 K and constant pressure (NPT ensemble) to account for volume changes due to fluctuations of homology models in solution.

Interaction energies were calculated considering the internal energy obtained with the specified force field, as well as the electrostatic and Van der Waals solvation energy obtained. The electrostatic solvation energy estimates the interaction energy between the solvent and the solute by treating the solvent as a continuum without explicit solvent molecules. A first-order boundary element approximation to the solvation energy was used. Van der Waals solvation energy was calculated as a function of the solute's solvent accessible surface area [48]. The entropic cost of fixing the ligand in the binding site is almost impossible to calculate accurately, but fortunately not needed since it mainly depends on characteristics that are constant during the simulation (ligand and protein size, side-chains on the surface etc.). The entropic component is thus a constant factor that can be omitted.  The more positive the interaction energy, the more favorable is the interaction in the context of the chosen force field.

### Enzyme isolation and characterization

*Aspergillus oryzae *strain CCF1066 (Czech Collection of Fungi, Faculty of Science, Charles University in Prague) was grown as described in [17]. Ammonium sulfate enzyme precipitate was purified by hydrophobic chromatography on Phenyl-Sepharose 6 Fast Flow (Amersham) using elution with the reversed ammonium sulfate gradient (0.6 M to 0 M in 20 mM sodium phosphate buffer pH6.8). Partially purified enzyme was concentrated and dialyzed against 20 mM sodium citrate buffer pH3.5. The enzyme was then purified on SP-Sepharose Fast Flow (Amersham) using elution with sodium chloride gradient (0 to 1 M). The enzyme was concentrated, dialyzed against 20 mM piperazine-HCl pH5.4, and purified on MonoQ HR 10/10 column (Amersham) eluted with sodium chloride gradient (0 to 0.3 M). The enzyme was concentrated to approx. 10 mg/mL, and stored in the stabilization buffer composed of 0.5 M ammonium sulphate in 50 mM citrate buffer pH5.0. Immediately before the spectroscopic measurements, the enzyme was transferred to 50 mM bis-Tris buffer pH5.0. The purity of the enzyme was checked by SDS-PAGE, and the concentration was determined by [49].

### Determination of enzymatic activity

Enzymatic activity was measured using 4-nitrophenyl-2-acetamido-2-deoxyglucopyranoside at either saturating concentration (5 mM, used for most determinations), or at concentrations around K_m _(for the evaluation of enzymatic parameters) according to the procedure of [50].

### Analysis of individual sites of *N*-glycosylation

Occupancy of sites of *N*-glycosylation was determined as described previously [23] by comparison of measured masses of peptides corresponding to the sites of glycosylation. The peptides were generated after SDS-PAGE separation by means of *in gel *digestion with sequencing grade trypsin (Promega), or sequencing grade Asp-*A *protease (Roche) either in normal or isotopic water H_2_^18^O (Fluka). The peptides were extracted from the gel, desalted and concentrated with C-18 microcolumn (ZipTip C18, Millipore) and analyzed by MS. To analyze individual sites of *N*-glycosylation, the glycoprotein was digested with trypsin, and glycopeptides were captured on concanavalin *A *– Sepharose resin (Amersham), washed, and eluted with 0.01 M Tris-HCl, pH8.0, 0.15 M NaCl and 0.3 M D-mannose (Sigma). Individual glycopeptides were separated by reverse-phase chromatography on the Vydac C-18 column (Dionex), equilibrated in 0.1% trifluoroacetic acid, and eluted by acetonitrile gradient to 70% over 120 min. The glycopeptides were identified by MALDI MS measurements in their native and or deglycosylated state. Glycans released from glycopeptides or from the intact protein with PNGase F (New England BioLabs) in 50 mM sodium bicarbonate pH8.0 were desalted on mini-columns (10 μL of bead volume) filled with non-porous graphitized carbon [51], and separated by HPAEC-PAD (DX500, Dionex) on Carbopac PA100 column. Elution was performed by 5 min isocratic step with 0.1 M NaOH (solvent A) followed by linear gradient from 0 to 35% of 0.1 M NaOH, 0.6 M NaOAc (solvent B) over 48 min and another linear gradient to 100% solvent B within 10 min at the flow rate 1 mL/min. The collected peaks were desalted (as above) and analysed by MALDI MS. For further characterization of the isolated oligosaccharides, the digestion with α-mannosidase was performed using 5 U/mL of the enzyme in 50 mM sodium citrate buffer pH5.0 [52].

### Enzymatic deglycosylation under native conditions

100 μg of β-*N*-acetylhexosaminidase was fully deglycosylated either in 100 μl of 50 mM ammonium bicarbonate pH8.0 using 500 units of *N*-glycanase (New England BioLabs), or in 100 μL of 50 mM citrate buffer pH5.0 using 500 units of EndoH (New England BioLabs). The reaction was carried out for 16 h at 37°C.

### pH stability test

pH stability test of the native and deglycosylated enzyme was performed as follows: 1 μL of the stock solution of enzyme was mixed with 9 μL of 50 mM sodium citrate buffer pH2.5, 3.0, 3.5, 4.0, or 4.5, or with 9 μL of 50 mM sodium phosphate buffer pH5.5, 6.0, 6.5, or 7.0, or 50 mM Tris-HCl buffer pH7.5, 8.0, 8.5, or 9.0. These mixtures were incubated for 16 h at 4°C, diluted with 40 μL of the substrate solution (50 mM sodium citrate pH5.0 with 5 mM substrate), and the enzymatic activity was determined as described in [50].

### FTIR spectroscopy

Infrared spectra of protein samples (9.5 mg/mL native and 8.5 mg/mL deglycosylated in 50 mM bis-Tris, pH5.0) were recorded in CaF_2 _10 μm BioCell™ (BioTools) at room temperature with a Bruker IFS-66/S FTIR spectrometer using a standard source, a KBr beamsplitter and an MCT detector. 4000 scans were collected with 4 cm^-1 ^spectral resolution and Happ-Genzel apodization function. Spectral contribution of a buffer in carbonyl stretching region was corrected following the standard algorithm [53].

### Raman spectroscopy

Raman spectra were recorded in a standard 90° geometry on a multichannel instrument based on a 600-mm single spectrograph (Monospec 600, Hilger & Watts) with a 1200-grooves/mm grating and a liquid N_2_-cooled CCD detection system (Princeton Instruments) having 1024 pixels along dispersion axis. A holographic notch-plus filter (Kaiser Optical Systems) was used to remove elastically scattered light. The effective spectral slit width was set to ~5 cm^-1^. Samples were excited with 514.5 nm/50 mW line of an Ar^+ ^laser Innova 300 (Coherent). Calibrated wavenumber scale by Ar^+ ^plasma lines was accurate to ± 1 cm^-1^.

Measurements were made on protein samples (8–13 mg/mL in 50 mM bis-Tris, pH5.0) in a 10-μL capillary microcell. Spectra, measured at 4°C, were averaged from 150 exposures of 120 s. Spectra were treated according to [54], then they were smoothed using 9-point Savitsky-Golay algorithm and normalized to the 1447 cm^-1 ^δCH_2 _band as an internal standard.

## Abbreviations

DTT, dithiothreitol; EDC, 1-ethyl-3-(3'-dimethylaminopropyl)-carbodiimide hydrochloride; FTIR, Fourier transform infrared; GalNAc, *N*-Acetyl-*D*-galactosamine; GlcNAc, *N*-Acetyl-*D*-glucosamine; HPAEC-PAD, high-performance anion exchange chromatography with pulsed amperometric detection; HPLC, high performace liquid chromatography; LSA, least-squares analysis; MALDI, matrix-assisted laser desorption/ionization; ManNAc, *N*-Acetyl-*D*-mannosamine; MD, molecular dynamics; MS, mass spectrometry; PNGase F, peptide-N4-(*N*-acetyl-β-*D*-glucosaminyl) asparagine amidase F; PSD, post-source decay; RESP, restrained electrostatic potential; SDS, sodium dodecyl sulfate; SDS-PAGE, SDS polyacrylamide gel electrophoresis.

## Authors' contributions

RE was responsible for all the work on sequence alignments, molecular modeling, and molecular dynamics studies and performed most of it. MK built up the ligand structure and supervised the docking, VK jr., KH and VB performed all the IR and Raman spectroscopy measurements and the data interpretation. KH was also responsible for the hexosaminidase production. PN, PP and PM were responsible for mass spectrometry measurements, and solved the disulfide bonds arrangement, NK performed the computational glycosylation and the binding energy calculations and OP and JS were responsible for the determination of hexosaminidase glycosylation and performed the deglycosylation studies. HR took part in the enzyme purification and primary structure determination, VK conceived the study and provided the strains and advice on hexosaminidase production, KB was responsible for the overall coordination of the project and produced the first draft of the manuscript, and was responsible for hexosaminidase purification. All authors read and approved the final manuscript.

## Supplementary Material

Additional file 1 **Molecular dynamics simulation of β-*N*-hexosaminidase with bound chitobiose**. The movie shows 1 ns of simulation of the equilibrated structure with chitobiose docked into the active site (non-reducing sugar: green; reducing sugar: white). Additionally, the catalytic amino acid Glu^346 ^and amino acids Asp^345 ^(pink) and Trp^482 ^(orange) are shown as stick models. The disulfide bridge connecting the large flexible loop (magenta) is shown in yellow. Click here for file

## References

[B1] Gooday GW, Zhu WY, O'Donell RW (1992). What are the roles of chitinases in the growing fungus?. FEMS Microbiol Lett.

[B2] Bulawa CE (1993). Genetics and molecular biology of chitin synthesis in fungi. Annu Rev Microbiol.

[B3] Cheng Q, Li H, Merdek K, Park JT (2000). Molecular characterization of the β-*N*-acetylglucosaminidase of Escherichia coli and its role in cell wall recycling. J Bacteriol.

[B4] Cohen E (2001). Chitin synthesis and inhibition: a revisit. Pest Manag Sci.

[B5] Mahuran DJ (1999). Biochemical consequences of mutations causing the GM2 gangliosidoses. Biochim Biophys Acta.

[B6] Křen V, Ščigelová M, Přikrylová V, Havlíček V, Sedmera P (1994). Enzymatic-synthesis of β-*N*-acetylhexosaminides of ergot alkaloids. Biocatalysis.

[B7] Rajnochová E, Dvořáková J, Huňková Z, Křen V (1997). Reverse hydrolysis catalysed by β-*N*-acetylhexosaminidase from *Aspergillus oryzae*. Biotechnol Lett.

[B8] Krist P, Herkommerová-Rajnochová E, Rauvolfová J, Semeňuk T, Vavrušková P, Pavlíček J, Bezouška K, Petruš L, Křen V (2001). Toward an optimal oligosaccharide ligand for rat natural killer cell activation receptor NKR-P1. Biochem Biophys Res Commun.

[B9] Weignerová L, Vavrušková P, Pišvejcová A, Thiem J, Křen V (2003). Fungal β-*N*-acetylhexosaminidases with high β-*N*-acetylgalactosaminidase activity and their use for synthesis of β-GalNAc-containing oligosaccharides. Carbohydr Res.

[B10] Tews I, Perrakis A, Oppenheimer A, Dauter Z, Wilson KS, Vorgias CE (1996). Bacterial chitobiase structure provides insight into catalytic mechanism and the basis of Tay-Sachs disease. Nat Struct Biol.

[B11] Prag G, Papanikolau Y, Tavlas G, Vorgaris CE, Petratos K, Oppenheim AB (2000). Structures of chitobiase mutants complexed with the substrate Di-*N*-acetyl-d-glucosamine: the catalytic role of the conserved acidic pair, aspartate 539 and glutamate 540. J Mol Biol.

[B12] Mark BL, Vocadlo DJ, Zhao D, Knapp S, Withers SG, James MNG (2001). Crystallographic evidence for substrate-assisted catalysis in a bacterial β-hexosaminidase. J Biol Chem.

[B13] Williams SJ, Mark BL, Vocadlo DJ, James MNG, Withers SG (2002). Aspartate 313 in the *Streptomyces plicatus *hexosaminidase plays a critical role in substrate-assisted catalysis by orienting the 2-acetamido group and stabilizing the transition state. J Biol Chem.

[B14] Maier T, Strater N, Schuette CG, Klingenstein R, Sandhoff K, Saenger W (2003). The X-ray crystal structure of human β-hexosaminidase B provides new insights into Sandhoff disease. J Mol Biol.

[B15] Huňková Z, Křen V, Ščigelová M, Weignerová L, Scheel O, Thiem J (1996). Induction of β-*N*-acetylhexosaminidase in *Aspergillus oryzae*. Biotechnol Lett.

[B16] Aspergillus oryzae beta-N-acetylhexosaminidase precursor (hexA) gene, complete cds. http://www.ncbi.nlm.nih.gov/entrez/viewer.fcgi?db=nuccore&id=29242776.

[B17] Hušáková L, Herkommerová-Rajnochová E, Semeňuk T, Kuzma M, Rauvolfová J, Přikrylová V, Ettrich R, Plíhal O, Bezouška K, Křen V (2003). Enzymatic discrimination of 2-acetamido-2-deoxy-D-mannopyranose-containing disaccharides using β-*N*-acetylhexosaminidases. Adv Synth Catal.

[B18] Mark BL, Vocadlo DJ, Zhao D, Knapp S, Withers SG, James MNG (2001). Biochemical and structural assessment of the 1-*N*-azasugar GalNAc-isofagomine as a potent family 20 β-*N*-acetylhexosaminidase inhibitor. J Biol Chem.

[B19] Mark BL, Mahuran DJ, Cherney MM, Zhao D, Knapp S, James MNG (2003). Crystal structure of human β-hexosaminidase B: understanding the molecular basis of Sandhoff and Tay-Sachs disease. J Mol Biol.

[B20] Plíhal O, Sklenář J, Kmoníčková J, Man P, Pompach P, Havlíček V, Křen V, Bezouška K (2004). *N*-glycosylated catalytic unit meets *O*-glycosylated propeptide: complex protein architecture in a fungal hexosaminidase. Biochem Soc Trans.

[B21] Lemieux MJ, Mark BL, Cherney MM, Withers SG, Mahuran DJ, James MNG (2006). Crystallographic structure of human β-hexosaminidase A: interpretation of Tay-Sachs mutations and loss of G_M2 _ganglioside hydrolysis. J Mol Biol.

[B22] Bařinka C, Šácha P, Sklenář J, Man P, Bezouška K, Slusher BS, Konvalinka J (2004). Identification of the *N*-glycosylation sites on glutamate carboxypeptidase II necessary for proteolytic activity. Protein Sci.

[B23] Gonzalez J, Takao T, Hori H, Besada V, Rodriguez R, Padron G, Shimonishi Y (1992). A method for determination of *N*-glycosylation sites in glycoproteins by collision-induced dissociation analysis in fast atom bombardment mass spectrometry: identification of the positions of carbohydrate-linked asparagine in recombinant alpha-amylase by treatment with peptide-*N*-glycosidase *F *in ^18^O-labeled water. Anal Biochem.

[B24] Novák P, Man P, Pompach P, Hofbauerová K, Bezouška K (2006). Straightforward Determination of Disulfide Linkages in Proteins: The Case of β-*N*-acetyl-Hexosaminidase from *Aspergillus oryzae*. Proceedings of the ASMS Conference on Mass Spectrometry and Allied Topics.

[B25] Arrondo JLR, Goñi FM (1999). Structure and dynamics of membrane proteins as studied by infrared spectroscopy. Prog Biophys Mol Biol.

[B26] Fabian H, Mäntele W, Chalmers JM, Griffiths PR (2002). Handbook of Vibrational Spectroscopy.

[B27] Yamada N, Ariga K, Naito M, Matsubara K, Koyama E (1998). Regulation of β-sheet structures within amyloid-like β-sheet assemblage from tripeptide derivatives. J Am Chem Soc.

[B28] Van Wart HE, Scheraga HA (1986). Agreement with the disulfide stretching frequency-conformation correlation of Sugeta, Go, and Miyazawa. Proc Natl Acad Sci USA.

[B29] Siamwiza MN, Lord RC, Chen MC, Takamatsu T, Harada I, Matsura H, Shimanouchi T (1975). Interpretation of the doublet at 850 and 830 cm^-1 ^in the Raman spectra of tyrosyl residues in proteins and certain model compounds. Biochemistry.

[B30] Ettrich R, Brandt W, Kopecký V, Baumruk V, Hofbauerová K, Pavlíček Z (2002). Study of chaperone-like activity of human haptoglobin: conformational changes under heat shock conditions and localization of interaction sites. Biol Chem.

[B31] Joseph D, Petsko GA, Karplus M (1990). Anatomy of a conformational change: hinged "lid" motion of the triosephosphate isomerase loop. Science.

[B32] Pakhomova S, Kobayashi M, Buck J, Newcomer ME (2001). A helical lid converts a sulfotransferase to a dehydratase. Nat Struct Biol.

[B33] Bustos-Jaimes I, Sosa-Peinado A, Rudino-Pinera E, Horjales E, Calcagno ML (2002). On the role of the conformational flexibility of the active-site lid on the allosteric kinetics of glucosamine-6-phosphate deaminase. J Mol Biol.

[B34] Brocca S, Secundo F, Ossola M, Alberghina L, Carrera G, Lotti M (2003). Sequence of the lid affects activity and specificity of Candida rugosa lipase isoenzymes. Protein Sci.

[B35] Pfeiffer G, Strube KH, Schmidt M, Geyer R (1994). Glycosylation of two recombinant human uterine tissue plasminogen activator variants carrying an additional *N*-glycosylation site in the epidermal-growth-factor-like domain. Eur J Biochem.

[B36] Hogg T, Kutá-Smatanová I, Bezouška K, Ulbrich N, Hilgenfeld R (2002). Sugar-mediated lattice contacts in crystals of a plant glycoprotein. Acta Crystallogr D Biol Crystallogr.

[B37] Berman HM, Westbrook J, Feng Z, Gilliland G, Bhat TN, Weissig H, Shindyalov IN, Bourne PE (2000). The Protein Data Bank. Nucl Acids Res.

[B38] Guex N, Peitsch MC (1997). SWISS-MODEL and the Swiss-PdbViewer: an environment for comparative protein modeling. Electrophoresis.

[B39] Thompson JD, Gibson TJ, Plewniak F, Jeanmougin F, Higgins DG (1997). The CLUSTAL_X windows interface: flexible strategies for multiple sequence alignment aided by quality analysis tools. Nucl Acids Res.

[B40] Sali A, Blundell TL (1993). Comparative protein modelling by satisfaction of spatial restraints. J Mol Biol.

[B41] Laskowski RA, McArthur MW, Moss DS, Thornton JM (1993). PROCHECK – a program to check the stereochemical quality of protein structures. J Appl Crystallog.

[B42] Berendsen HJC, van der Spoel D, van Drunen R (1995). GROMACS: a message-passing parallel molecular dynamics implementation. Comput Phys Commun.

[B43] Lindahl E, Hess B, van der Spoel D (2001). GROMACS 3.0: A package for molecular simulation and trajectory analysis. J Mol Modell.

[B44] Berendsen HJC, Postma JPM, van Gunsteren WF, DiNola A, Haak JR (1984). Molecular-dynamics with coupling to an external bath. J Chem Phys.

[B45] Bohne A, Lang E, von der Lieth CW (1998). W3-SWEET: Carbohydrate modeling by Internet. J Mol Model.

[B46] Krieger E, Darden T, Nabuurs SB, Finkelstein A, Vriend G (2004). Making optimal use of empirical energy functions: force-field parameterization in crystal space. Proteins.

[B47] Essman U, Perera L, Berkowitz ML, Darden T, Lee H, Pedersen LG (1995). A smooth particle mesh Ewald method. J Chem Phys.

[B48] Bultinck P, De Winter H, Langenaeker W, Tollenare J (2003). Computational medicinal chemistry for drug discovery.

[B49] Bradford MM (1976). A rapid and sensitive method for the quantitation of microgram quantities of protein utilizing the principle of protein-dye binding. Anal Biochem.

[B50] Li SC, Li YT (1970). Studies on the glycosidases of jack bean meal. 3. Crystallization and properties of β-*N*-acetylhexosaminidase. J Biol Chem.

[B51] Packer NH, Lawson MA, Jardine DR, Redmond JW (1998). A general approach to desalting oligosaccharides released from glycoproteins. Glycoconj J.

[B52] Harvey DJ (1999). Matrix-assisted laser desorption/ionization mass spectrometry of carbohydrates. Mass Spectrom Rev.

[B53] Dousseau F, Therrien M, Pézolet M (1989). On the spectral substraction of water from the FT-IR spectra of aqueous-solutions of proteins. Appl Spectrosc.

[B54] Williams RW (1986). Protein secondary structure analysis using Raman amide I and amide III spectra. Methods Enzymol.

[B55] Dousseau F, Pézolet M (1990). Determination of the secondary structure content of proteins in aqueous solutions from their amide I and amide II infrared bands. Comparison between classical and partial least-squares methods. Biochemistry.

